# Metabolic Glycoengineering in hMSC-TERT as a Model for Skeletal Precursors by Using Modified Azide/Alkyne Monosaccharides

**DOI:** 10.3390/ijms22062820

**Published:** 2021-03-10

**Authors:** Stephan Altmann, Jürgen Mut, Natalia Wolf, Jutta Meißner-Weigl, Maximilian Rudert, Franz Jakob, Marcus Gutmann, Tessa Lühmann, Jürgen Seibel, Regina Ebert

**Affiliations:** 1 Bernhard-Heine-Center for Locomotion Research, University of Würzburg, Friedrich-Bergius-Ring 15, 97076 Würzburg, Germany; stephan.altmann@klh.de (S.A.); j-meissner-weigl.klh@uni-wuerzburg.de (J.M.-W.); m-rudert.klh@uni-wuerzburg.de (M.R.); f-jakob.klh@uni-wuerzburg.de (F.J.); 2 Institute of Organic Chemistry, University of Würzburg, Am Hubland, 97074 Würzburg, Germany; juergen.mut@uni-wuerzburg.de (J.M.); natalia.wolf@uni-wuerzburg.de (N.W.); juergen.seibel@uni-wuerzburg.de (J.S.); 3 Institute of Pharmacy and Food Chemistry, University of Würzburg, Am Hubland, 97074 Würzburg, Germany; Marcus.Gutmann@uni-wuerzburg.de (M.G.); tessa.luehmann@uni-wuerzburg.de (T.L.)

**Keywords:** hMSC-TERT, metabolic glycoengineering, glycocalyx, modified monosaccharides, click chemistry

## Abstract

Metabolic glycoengineering enables a directed modification of cell surfaces by introducing target molecules to surface proteins displaying new features. Biochemical pathways involving glycans differ in dependence on the cell type; therefore, this technique should be tailored for the best results. We characterized metabolic glycoengineering in telomerase-immortalized human mesenchymal stromal cells (hMSC-TERT) as a model for primary hMSC, to investigate its applicability in TERT-modified cell lines. The metabolic incorporation of *N*-azidoacetylmannosamine (Ac_4_ManNAz) and *N*-alkyneacetylmannosamine (Ac_4_ManNAl) into the glycocalyx as a first step in the glycoengineering process revealed no adverse effects on cell viability or gene expression, and the in vitro multipotency (osteogenic and adipogenic differentiation potential) was maintained under these adapted culture conditions. In the second step, glycoengineered cells were modified with fluorescent dyes using Cu-mediated click chemistry. In these analyses, the two mannose derivatives showed superior incorporation efficiencies compared to glucose and galactose isomers. In time-dependent experiments, the incorporation of Ac_4_ManNAz was detectable for up to six days while Ac_4_ManNAl-derived metabolites were absent after two days. Taken together, these findings demonstrate the successful metabolic glycoengineering of immortalized hMSC resulting in transient cell surface modifications, and thus present a useful model to address different scientific questions regarding glycosylation processes in skeletal precursors.

## 1. Introduction

Bone marrow-derived human mesenchymal stromal cells (hMSC) are a heterogeneous population of adult precursor cells, which have self-renewal and multilineage differentiation capacities. Minimal criteria to define hMSC in vitro comprise their plastic adherence, the expression of specific mesenchymal markers and the absence of hematopoietic markers, as well as their capacity to differentiate toward the osteogenic, adipogenic, and chondrogenic lineage [1,2]. The cultivation duration of primary hMSC is limited, because cells in culture undergo the process of replicative senescence [3]. This characteristic, as well as their heterogeneity, makes it difficult to use them for the establishment of standardized procedures. A possibility to overcome these problems is to use an appropriate cell line, for example telomerase-immortalized human mesenchymal stromal cells (hMSC-TERT). The overexpression of telomerase overrides replicative senescence and the cells display a high proliferation rate while maintaining their mesenchymal differentiation capacity [4]. Therefore, hMSC-TERT are a suitable in vitro model for primary hMSC and can be used to establish standardized methods.

As the outermost layer of the cell, the glycocalyx is a highly versatile network consisting mainly of proteoglycans [5] and glycoproteins covering the membrane. The glycocalyx exhibits different important functions, e.g., from being a physical barrier regulating endocytotic processes [6,7,8], mediating immunogenicity [9,10,11,12], cell adhesion [13,14,15] and signaling [16], to the transduction of shear stress toward the cytoskeleton [17,18,19]. The functions are mainly related to interactions with the cell environment, which also defines its characteristics such as molecular composition, organization [20,21], and thickness [22]. Due to its biochemical importance and its ease of accessibility for experimental manipulations, the cell glycocalyx offers great potential as a target for questions in basic and biomedical research. The method of choice here is metabolic glycoengineering, representing an easy-to-use and versatile tool for the investigation or modification of glycosylated macromolecules such as proteins or glycans [23]. For this purpose, cells are fed with monosaccharides bearing unnatural functional groups such as azides or alkynes, resulting in their incorporation into macromolecules as part of the glycosylation process [24]. While some of them remain in the cytosol, others are transferred to the cell membrane and become parts of the glycocalyx [25]. Here, these modified monosaccharides are accessible for click chemistry, which allows the introduction of specific molecules dependent on the respective scientific question. Although the glycosylation process cannot be controlled per se, parameters such as the incorporation efficiency or cell viability can be optimized by adjusting the methodic conditions [26]. This is even more important when glycoengineered cells are used for in vivo purposes, in which the quality of the glycoengineering affects subsequent cell behavior in specific environments or situations.

In cell therapy-based research, glycoengineering is applied to temporarily enhance specific cell features. The transient surface expression of an E-selectin ligand could increase the osteotropism of systemically administered hMSC in mice [27], and the glycoengineering of human adipose-derived stromal cells (hASC) enabled the monitoring and tracking of cell fate in mice [28]. Conjugation of glycoengineered hMSC with paclitaxel-loaded nanoparticles and its subsequent systemically administration in mice led to enhanced tumor homing and its growth inhibition [26], thus proving a therapeutic approach. Additionally, topics such as glycomics [29], molecular imaging, immunomodulation, or extracellular communication, e.g., by small molecules or antibody-based modulators of glycosyltransferases and glycosidases [30,31] can be addressed with this technique. Many of the cell functions that are mediated by glycans and glycosylation states are involved in cell differentiation and development, as well as in stem cells of various differentiation stages and origins [32,33,34]. It has been reported that glycosylation impacts osteogenic differentiation of skeletal precursors; hence, metabolic glycoengineering of skeletal precursors may offer an excellent possibility to further dissect the influence of glycosylation on bone formation and regeneration and open up new therapeutic targets [35,36]. Once the altered cells reach their destiny, it is desirable that they return to a native cell state omitting any artificial modifications. On the contrary, a certain stability of these alterations is necessary to define the time window for further processing of glycoengineered cells, which is of interest for research in tissue regeneration and engineering. The attachment capability of glycoengineered macrophages resulted in a better in situ cellularization of a tissue scaffold implanted in mice [37]. Additionally, our research, embedded in the biofabrication field, aims to improve the cell adherence after bioprinting and also the cell stability by modifying the cell glycocalyx.

In this study, we investigated the incorporation of *N*-azidoacetylglucosamine (Ac_4_GlcNAz), *N*-azidoacetylgalactosamine (Ac_4_GalNAz), *N*-azidoacetylmannosamine (Ac_4_ManNAz), and *N*-alkyneacetylmannosamine (Ac_4_ManNAI) into the glycocalyx of hMSC-TERT and their functional impact on the cells as the first step toward the application in biofabrication processes. hMSC-TERT were used as a model for primary bone marrow-derived hMSC to overcome their heterogeneity and donor variabilities. Besides the comparative analysis of the incorporation efficiency, we looked for the long-term stability of the introduced modified monosaccharides. The complementary assessment of cell viability, differentiation potential, and gene expression shall provide insights into the question of how the cells adapt to the presence of modified monosaccharides. Finally, the results will help to evaluate if and how far metabolic glycoengineering can be applied to TERT-modified skeletal precursors.

## 2. Results

### 2.1. Impact of Click Reagents on the Cell Viability

**Figure 1:**
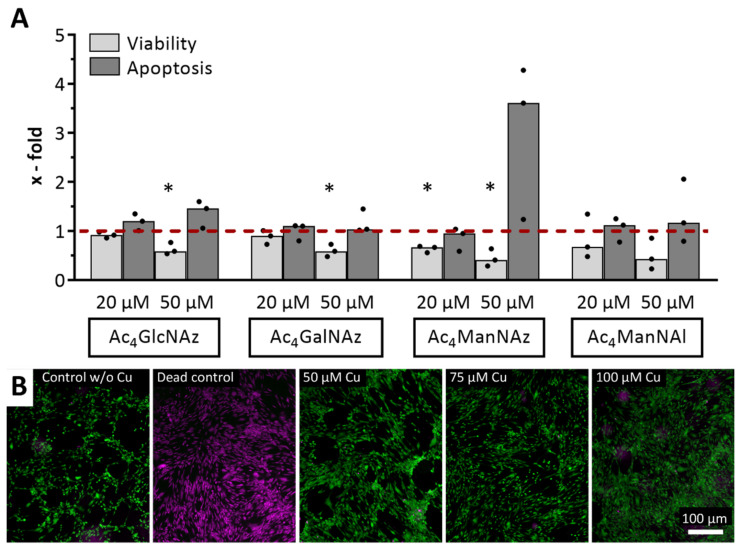
Cell viability of telomerase-immortalized human mesenchymal stromal cells (hMSC-TERT) after treatment with click reagents. (**A**) Viability and apoptosis rates after 72 h incubation with different modified monosaccharides relative to DMSO-treated control (dashed line). Datasets (median with individual values) were tested for significance using two-tailed paired *t*-test (*p* < 0.05 depicted as *). (**B**) Live/dead staining after 5 min incubation with Cu(I)-containing click reaction mixture. Green: fluorescence signal of living cells, magenta: fluorescence signal of dead cells. Representative images of three independent experiments are shown.

Prior to the characterization of metabolic glycoengineering, we tested the impact of click reagents on the cell features to define the optimal experimental conditions. All four investigated modified monosaccharides showed only slightly increased apoptosis rates, except Ac_4_ManNAz, which was 3.6-fold higher than the control, and significantly (*p* = 0.03) decreased viability rates (40 to 60%) at 50 µM concentrations (Figure 1A). The results for the 20 µM concentrations showed viability rates above 90% for Ac_4_GlcNAz and Ac_4_GalNAz and 67% for both mannose sugars, while there was no remarkably increased apoptosis.

The copper(I)-catalyzed azide-alkyne cycloaddition (CuAAC) click reaction is catalyzed by Cu(I) ions, which are stabilized by the THPTA ligand. Although the five-fold ligand amount should bind all free Cu(I) species, we analyzed whether potential residual free cytotoxic Cu(I) species have an impact on cell viability [38]. Live/dead staining showed mostly living cells directly after incubation with the click reaction mixture (containing different concentrations of CuSO_4_, the respective five-fold concentration of THPTA, and 2.5 mM sodium ascorbate), with almost no dead cells, for all chosen concentrations (Figure 1B). Despite a potentially higher reaction efficiency, we used 50 µM for all further experiments to keep Cu(I) ion-mediated cytotoxicity as low as possible.

### 2.2. Evaluation of Different Modified Monosaccharides Regarding Their Incorporation and Retention in the Glycocalyx

To assess the metabolic glycoengineering of hMSC-TERT, we tested all four modified monosaccharides in two commonly used concentrations (20 and 50 µM) for their potential to incorporate into the glycocalyx by clicking a detectable fluorescent dye using the CuAAC click reaction.

**Figure 2:**
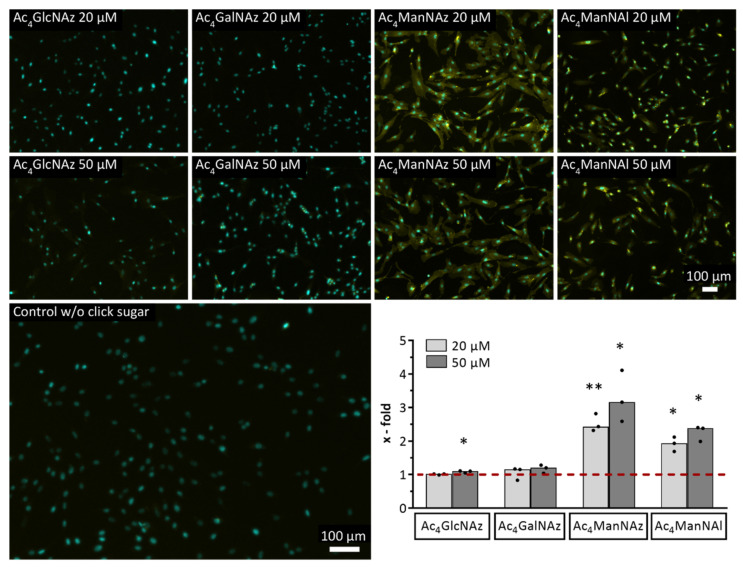
Incorporation of four different modified monosaccharides into the glycocalyx of hMSC-TERT after 72 h incubation. Yellow: fluorescence signal of Cy 3/5 after copper(I)-catalyzed azide-alkyne cycloaddition (CuAAC) click reaction, cyan: fluorescence signal of cell nuclei. Representative fluorescence images of three independent experiments are shown. The diagram shows the quantitative analysis of fluorescence micrographs from three independent experiments. Cy 3/5 fluorescence signal as a mean grey value is depicted relative to the DMSO-treated control (dashed line). Datasets (median with individual values) were tested using two-tailed paired *t*-test for significance (*p* < 0.05 depicted as *, *p* < 0.01 depicted as **).

Although there was no detectable fluorescence upon incubation with Ac_4_GlcNAz or Ac_4_GalNAz compared to the DMSO-treated control, the mannose sugars showed a 2.4- to 3.2-fold (Ac_4_ManNAz) or a 1.9- to 2.4-fold (Ac_4_ManNAl) higher fluorescence intensity (Figure 2). Therefore, we decided to use both mannose sugars for all further experiments and to refrain from applying Ac_4_GlcNAz or Ac_4_GalNAz.

The cell glycocalyx is subject to permanent turnover and remodeling due to intrinsic biosynthetic processes and extrinsic influences such as shear forces [39]; therefore, we wanted to know how stable the chemical modifications, that were introduced by click reactions, were over time. For that reason, we evaluated the glycocalyx state at several time points up to 6 d.

**Figure 3:**
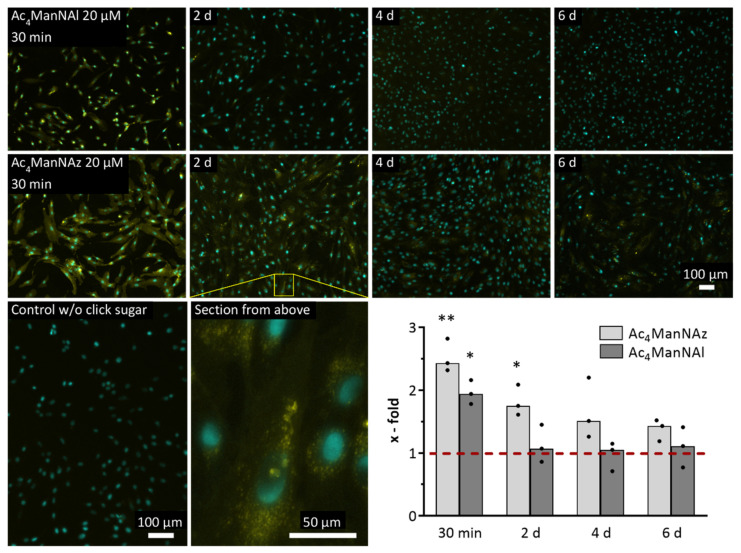
Temporal course of the decline of glycocalyx modification in hMSC-TERT after 72 h incubation with Ac_4_ManNAz or Ac_4_ManNAl. Yellow: fluorescence signal of Cy 3/5 after CuAAC click reaction, cyan: fluorescence signal of cell nuclei. Representative fluorescence images of three independent experiments are shown. The diagram shows the quantitative analysis of fluorescence micrographs from three independent experiments. Cy 3/5 fluorescence signal as a mean grey value is depicted relative to the DMSO-treated control (dashed line). Datasets (median with individual values) were tested for significance using two-tailed paired *t*-test (*p* < 0.05 depicted as *, *p* < 0.01 depicted as **).

Although both mannose sugars revealed an initially strong decrease in fluorescence intensity after 2 d (48% for Ac_4_ManNAz and 93% for Ac_4_ManNAl), their subsequent temporal course was different (Figure 3). In contrast to the missing fluorescence of Ac_4_ManNAl, the azido mannose still showed a fluorescence signal, which continuously and evenly declined until day six (from 1.8-fold to 1.4-fold). Interestingly, cells incubated for 2 d or longer revealed small focal aggregates within the cytoplasm (section of Ac_4_ManNAz 20 µM 2 d in Figure 3).

### 2.3. Impact of Modified Monosaccharides on Mesenchymal Differentiation and Gene Regulation

**Figure 4:**
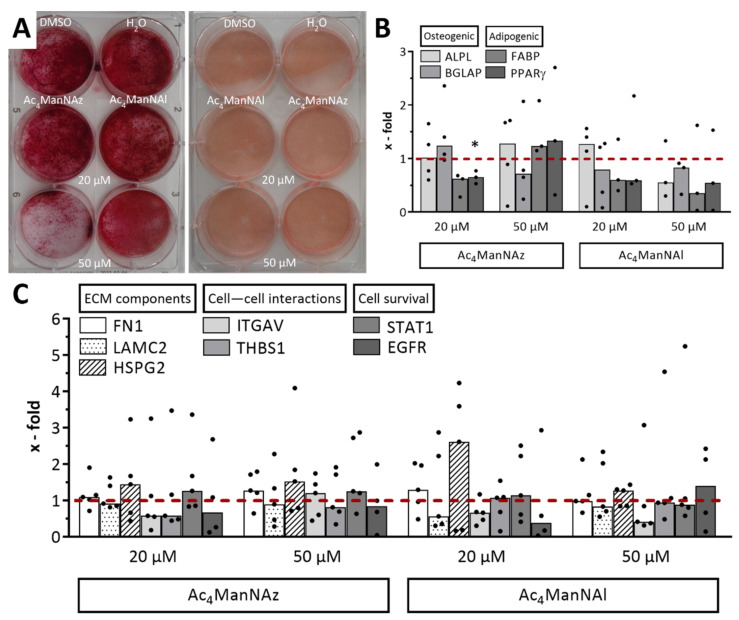
Differentiation capacity and gene expression of hMSC-TERT after 72 h incubation with Ac_4_ManNAz or Ac_4_ManNAl. (**A**) Alizarin Red and Oil Red staining after two weeks of osteogenic or adipogenic differentiation. Representative images of three independent experiments are shown. (**B**) Gene expression analysis of alkaline phosphatase (ALPL) and bone gamma carboxyglutamate protein (BGLAP) (osteogenic markers) as well as fatty acid binding protein (FABP) and peroxisome proliferator activated receptor gamma (PPARγ) (adipogenic markers) relative to DMSO-treated control (dashed line). Datasets (median with individual values) were tested for significance using two-tailed paired *t*-test (*p* < 0.05 depicted as *). (**C**) Gene expression analysis of fibronectin (FN1), laminin subunit gamma 2 (LAMC2), heparan sulfate proteoglycan (HSPG2), integrin subunit alpha V (ITGAV), thrombospondin 1 (THBS1), signal transducer and activator of transcription 1 (STAT1), and epidermal growth factor receptor (EGFR) relative to DMSO-treated control (dashed line). Extracellular matrix (ECM). Datasets (median with individual values) were tested for significance using two-tailed paired *t*-test.

Sugars are involved in many metabolic pathways; therefore, we analyzed the influence of Ac_4_ManNAz or Ac_4_ManNAl on the mesenchymal differentiation potential of hMSC-TERT as well as their impact on gene expression. Alizarin Red staining revealed the formation of calcium phosphate aggregates for both modified monosaccharides (Figure 4A left) after two weeks of osteogenic differentiation (it was less pronounced for Ac_4_ManNAz 50 µM). Oil Red staining resulted in evenly distributed lipid droplet formations in all samples (Figure 4A right) under adipogenic conditions. Gene expression analyses of differentiation marker genes (alkaline phosphatase (ALPL) and bone gamma carboxyglutamate protein (BGLAP) (osteogenic markers), as well as fatty acid binding protein (FABP) and peroxisome proliferator activated receptor gamma (PPARγ) (adipogenic markers)) showed fold changes in the range from 0.4 to 1.3 after 72 h incubation with modified monosaccharides and subsequent differentiation (Figure 4B). In detail, stimulation with Ac_4_ManNAz led to an increase of up to 1.3-fold for BGLAP (20 µM), ALPL, and both adipogenic marker genes (50 µM), as well as for ALPL after incubation with 20 µM Ac_4_ManNAl. On the contrary, stimulation with Ac_4_ManNAl resulted in decreased gene expression after osteogenic differentiation (0.6- to 0.8-fold for ALPL and BGLAP at 50 µM, or the latter at 20 µM) or adipogenic differentiation (0.4- to 0.6-fold for FABP and PPARγ at both concentrations). Additionally, stimulation with Ac_4_ManNAz (20 µM) revealed a decrease of 0.6- to 0.7-fold for both adipogenic marker genes (20 µM) or BGLAP at 50 µM. Overall, the fold changes were scattered in all but two cases.

In addition, a set of seven genes responsible for different cell functions were analyzed for fold changes after incubation with Ac_4_ManNAz or Ac_4_ManNAl. Functional areas were represented by fibronectin (FN1), laminin subunit gamma 2 (LAMC2), and heparan sulfate proteoglycan (HSPG2) linked to extracellular matrix components, integrin subunit alpha V (ITGAV) and thrombospondin 1 (THBS1) linked to cell-cell interactions, as well as signal transducer and activator of transcription 1 (STAT1) and epidermal growth factor receptor (EGFR) linked to cell survival and signal transduction. Although most conditions did not result in significant fold changes compared to the DMSO-treated control, the expression of three genes was slightly increased by using both modified monosaccharides (Figure 4C). In detail, FN1 showed a slight increase of 1.3-fold (Ac_4_ManNAz 50 µM and Ac_4_ManNAl 20 µM), HSPG2 an increase of 1.4- to 1.5-fold (Ac_4_ManNAz) or 2.6-fold for Ac_4_ManNAl 20 µM, and STAT1 an increase of 1.3-fold (Ac_4_ManNAz). Additionally, ITGAV (1.2-fold for Ac_4_ManNAz) and EGFR (1.4-fold for Ac_4_ManNAl) were slightly increased at 50 µM. Decreased gene expressions were seen for LAMC2 (0.9-fold for Ac_4_ManNAz and 0.6 to 0.8-fold for Ac_4_ManNAl), ITGAV (0.6 to 0.7-fold at 20 µM or 0.4-fold for Ac_4_ManNAl 50 µM), and THBS1 (0.6 to 0.8-fold for Ac_4_ManNAz). Furthermore, gene expression of STAT1 with 0.9-fold for Ac_4_ManNAl 50 µM as well as EGFR (0.7- to 0.8-fold for Ac_4_ManNAz or 0.4-fold as a consequence of incubation with Ac_4_ManNAl used at 20 µM) were diminished. Although the data showed high variances, it should be noted that there were often grouped single values with one or two outliers representing natural biological variation.

## 3. Discussion

To set up the optimal conditions for metabolic glycoengineering prior to the click experiments, we assessed the impact of modified monosaccharides on the phenotype of hMSC-TERT. Regarding viability, sugar-treated cells revealed decreased viability rates but no remarkably increased apoptosis rates, except partially for Ac_4_ManNAz at 50 µM (Figure 1), which would indicate potential cytotoxicity. Modulation of both intrinsic and extrinsic apoptosis pathways, such as via death receptors, cannot be excluded as a reason for this phenomenon, although the influence of glycans and glycosylation patterns on cell death induction is incompletely understood [40]. In 2004, Kim et al. showed a correlation between diminished cell growth and enhanced sialic acid production after the treatment of Jurkat cells with 500 µM Ac_4_ManNAc, while cell viability was similar to untreated cells [41]. These findings are supported by our data from modified *N*-acetylmannosamine (ManNAc) analogs using a different approach. Linked to the incorporation efficiencies, the decreased viability rates for both mannose sugars would correlate with the respective sugar concentration, because a higher concentration results in a higher incorporation efficiency (Figure 2). Despite no detection via click chemistry, we assume that the cells rather use the azido variants of *N*-acetylglucosamine (GlcNAc) and *N*-acteylgalactosamine (GalNAc) for *O*-linked GlcNAc processing [25]. This glycosylation type results in glycoproteins residing in the nucleus and the cytosol, which cannot be targeted by the alkyne-Cy3 dye during the click reaction, reflected by the absence of fluorescence signals (Figure 2). Regarding the click reaction, cell viability was high even at Cu concentrations up to 100 µM (Figure 1B), which can be explained by the short exposure time of 5 min with subsequent washing steps removing residual Cu(I) ions and the stable complexing through the ligand [42]. This short-time resistance against Cu(I) ions has already been shown in viability tests for NIH 3T3 fibroblasts and HEK293-F cells in 2016. In those experiments, the cells still showed a survival rate of approximately 80% after 10 min incubation with up to 200 µM Cu (both), and even after 20 min for the HEK293-F cells [43].

Additionally, we analyzed whether the stimulation of hMSC-TERT with modified monosaccharides has an impact on their mesenchymal differentiation capacity. The assessment of the osteogenic and adipogenic differentiation potential suggests no remarkable impairment after incubation with Ac_4_ManNAz or Ac_4_ManNAl. The Oil Red staining results revealed a highly regular lipid accumulation regardless of the test conditions (Figure 4A, right), and the Alizarin Red staining also revealed positive results for all conditions, confirming a successful mineralization as well (Figure 4A, left). The marker gene expression data showed no significant inhibition or increased fold changes for adipogenic and osteogenic marker genes after incubation with Ac_4_ManNAz at 20 µM, suggesting that the differentiation process was not severely impaired (Figure 4B). To obtain more insight into the molecular influence of both mannose sugars Ac_4_ManNAz and Ac_4_ManNAl, we analyzed their impact on gene expression in hMSC-TERT. Genes were selected according to a paper published by the group of Han et al. [44]. The authors showed that Ac_4_ManNAz, if applied in concentrations higher than 20 µM, led to a downregulation of genes related to one of the following four subgroups: cell adhesion, cytokine-cytokine receptor interaction, extracellular matrix (ECM)-receptor interaction, and cancer-related pathways. We selected seven genes from these groups and analyzed their expression in hMSC-TERT after 72 h incubation with Ac_4_ManNAz or Ac_4_ManNAl by qPCR. All examined gene expressions were affected by high variances for all conditions, suggesting no relevant biological conclusions for these datasets (Figure 4C). Considering that there were no obvious differences (also not as trend findings) deriving from the functional click group or the concentration, we assume that our data indicate no interference of Ac_4_ManNAz or Ac_4_ManNAl with transcriptional processes during protein biosynthesis in hMSC-TERT as opposed to that shown in endothelial progenitor cells.

Several modified monosaccharides exist and are used for metabolic glycoengineering; therefore, we tested Ac_4_GlcNAz, Ac_4_GalNAz, Ac_4_ManNAz, and Ac_4_ManNAl as alkyne analogs for their biochemical incorporation into the glycocalyx of hMSC-TERT. We could detect only Ac_4_ManNAz and Ac_4_ManNAl in the glycocalyx as part of glycans (Figure 2), however with a difference regarding their respective incorporation efficiency. Although the indirectly derived fold changes for both mannose sugars in the range of 1.9 to 2.3 seem to be small by numbers, we expect this level of incorporation efficiency to be sufficient for our planned functional modifications in the biofabrication field, which has to be confirmed by subsequent studies. Additionally, it is noteworthy that metabolic glycoengineering of adherent cells might generally result in lower modification degrees because the apical side of the cell layer is primarily exposed to the click reagents. We assume that the different incorporation efficiencies do not result from processes prior to the glycosylation because the uptake of the modified monosaccharides into the cell regulated by the diffusion rate and their subsequent enzymatic deacetylation by unspecific esterases should work for all four molecules. Therefore, we conclude similar intracellular sugar concentrations for subsequent glycosylation pathways. Even though an enzyme exists for the direct conversion of GlcNAc to ManNAc (*N*-Acylglucosamine 2-epimerase), the hMSC-TERT might use Ac_4_GlcNAz to fill the UDP-GlcNAc pool for the cytosolic *O*-GlcNAc glycosylation instead. GalNAc can be converted to UDP-GlcNAc via a three-step process; therefore, the similar absent incorporation of Ac_4_GalNAz into the glycocalyx might be a consequence [25]. By far the strongest fluorescence signals could be observed when using the mannose sugars. In contrast to the other two isomers, these monosaccharides are direct precursors of sialic acid, promoting its biosynthesis and related negative feedback loop [45,46]. Obviously, the glycosylation process does not only depend on the type of sugar which is used, but also on the cell type. In previous studies, we have shown that the glycoengineering of neuroblastoma (SK-N-MC) and osteosarcoma (U2OS) cell membranes resulted in the highest density of labeled glycans for GalNAz glycoconjugates, followed by ManNAz-derived glycoconjugates, while GlcNAz glycoconjugates showed the lowest density with a peculiar dependence on the investigated cell type [47]. In further studies, treatment of HEK 293 cells with Ac_4_GlcNAz [43] or Ac_4_GalNAz [48] resulted in a proper incorporation as seen in this study for the mannose sugars, while Ac_4_ManNAz showed different incorporation efficiencies for pancreatic cell lines SW1990 and PANC-C1 as well as CHO and Jurkat cells [49]. Moreover, quantification of whole cell sialic acid levels after incubation with different modified monosaccharides by Dold et al. [48] revealed sugar analog-dependent differences between HEK 293T and HeLa S3 cells, but also in the compartmentalization of the respective sialic acids between cell membranes and cytosol. Interestingly, a study from 2009 showed a higher conversion of Ac_4_ManNAl into the respective sialic acid than Ac_4_ManNAz in six different cell lines [50], but without any investigation regarding their compartmentalization. Taking into account the impact of the specific cell environment on the glycocalyx development in terms of physical interactions (higher shear forces by fluid flow as an example for endothelial cells [51]) and nutrition supply, these observations also suggest specific enzyme expression patterns in the sialic acid biosynthesis pathway leading to different metabolic fluxes.

After detecting the successful incorporation of modified monosaccharides, we wanted to know how long these introduced modifications persist in the glycocalyx, which will be important for the applications of glycoengineered cells. The microscopic results revealed an initial decrease in the fluorescence signal for both mannose sugars, meaning a loss of the introduced glycocalyx modifications (Figure 3). While these alterations disappeared after 2 d when using Ac_4_ManNAl, the fluorescence signal of the covalently bound alkyne-Cy3 slowly decreased over time, but was still detectable after 6 d. In previous studies with primary hMSC [26] or hASC [28], a constant decline of the fluorescence signal over a period of 7 d or even 14 d in the case of hASC could be observed, with almost no signal at the endpoint. With regard to our Ac_4_ManNAz data, it can be assumed that the modified hMSC-TERT will behave similarly and return to their native glycocalyx state after a while. The reason for the missing fluorescence signal after 2 d when using Ac_4_ManNAl is not obvious because both functional groups (azide and alkyne) are biological inert and small in terms of steric hindrance [52]. The only difference lies in their polarity; the azide exhibits delocated partial charges, which could interfere at least with enzymatic cleavage. Finally, the cells showed small focal signals (section of Ac_4_ManNAz 20 µM 2 d in Figure 3) after 2 d, which persisted until 6 d. These structures might be internalized glycoprotein fragments as part of salvage processes in the light of glycocalyx turnover, which was also suggested by Layek et al. [26].

Concerning the numerous application possibilities for metabolic glycoengineering due to the feasibility of introducing any target molecule into the cell glycocalyx, it is important to know which modified monosaccharides provide the best outcomes in terms of viability and incorporation efficiency. In particular, if animals or patients are involved in the study, the glycoengineering of cells needs to be optimized for the respective purposes. This study further elucidates the surprisingly variable glycosylation of glycocalyx-located proteins in dependence on the click group-bearing monosaccharide and the cell type. We could demonstrate the successful incorporation of modified monosaccharides into the glycocalyx of hMSC-TERT; therefore, further studies could focus on interesting applications for glycoengineered primary hMSC using hMSC-TERT as an easy-to-manage model before starting time-consuming experiments with primary cells or even animals.

## 4. Materials and Methods

### 4.1. Cell Culture

hMSC-TERT were established from a 33-year-old male donor by the group of Kassem (Odense, Denmark). These cells show high proliferation rates, while maintaining their mesenchymal differentiation capacity in vitro and in vivo [53,54]. Cells were cultured in Eagle’s Minimum Essential Medium (MEM) (Thermo Fisher Scientific, Waltham, MA, USA) supplemented with 10% (*v*/*v*) heat-inactivated fetal calf serum (FCS) (Bio & Sell, Ulm, Germany) [55], 50 µg mL^−1^ gentamicin sulfate, and 100 nM sodium selenite (both Sigma-Aldrich Chemie GmbH, Munich, Germany) [56] at 37 °C in a 95% humidified air and 5% CO_2_ atmosphere.

#### 4.1.1. Synthesis of 2-(*N*-4-Pentynoyl)-2-Deoxy-d-Mannopyranoside

**Figure 5:**
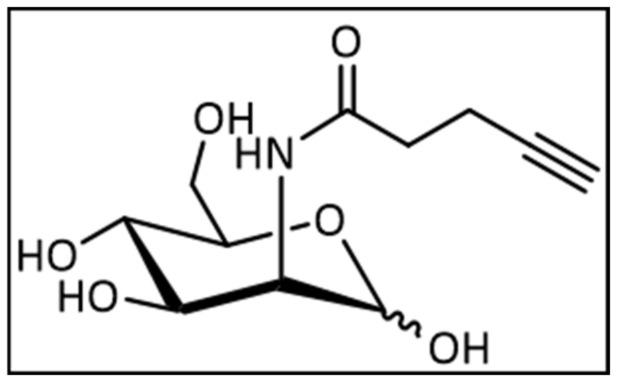
Structural formula of 2-(*N*-4-Pentynoyl)-2-Deoxy-d-Mannopyranoside.

The alkyne mannose was synthesized according to the literature [57], with slight modification. Briefly, 2-amino-2-deoxy-d-manno-pyranose hydrochloride (238 mg, 1.10 mmol, 1.00 eq.) was dissolved in dry methanol (12 mL). Na_2_CO_3_ (132 mg, 1.25 mmol, 1.13 eq.) and *O*-succinimide-4-pentynoic acid were added to the solution and the mixture was stirred at ambient temperature for 16 h. The solvent was evaporated and the crude product was purified by column chromatography (DCM:MeOH 9:1 → 8:2). Yield: 85% (243 mg, 937 µmol). Figure 5 is the structural formula of 2-(*N*-4-Pentynoyl)-2-Deoxy-d-Mannopyranoside.

The ^1^H-NMR of a major anomer (400 MHz, MeOD) (Supplementary Figure S1): 5.00 (d, ^3^*J* = 1.6 Hz, 1H, *H*-1), 4.30 (dd, ^3^*J* = 4.7 Hz, ^3^*J* = 1.6 Hz, 1H, *H*-2), 4.00 (dd, ^3^*J* = 9.7 Hz, ^3^*J* = 4.9 Hz, 1H, *H*-3), 3.87–3.75 (m, 3H, *H*-5, *H*-6), 3.58 (dd, ^3^*J* = 9.6 Hz, 1H, *H*-4), 2.49–2.46 (m, 3H, *H*-2′, *H*-3′), 2.27–2.25 (m, 1H, *H*-5′) ppm; ^13^C-NMR of a major anomer (100 MHz, MeOD) (Supplementary Figure S2): 174.71 (*C*-1′), 95.00 (*C*-1), 83.70 (*C*-4′), 73.44 (*C*-5), 70.62 (*C*-3), 70.24 (*C*-5′), 68.48 (*C*-4), 62.23 (*C*-6), 55.09 (*C*-2), 35.88 (*C*-2′), 15.63 (*C*-3′) ppm; HRMS (ESI): *m/z* calculated for C_11_H_17_NO_6_ [M-Cl^−^] 294.0750, measured 294.0758, (|Δ*m/z*| = 2.8 ppm).

#### 4.1.2. Synthesis of 2-(*N*-4-Pentynoyl)-2-Deoxy-(1,3,4,6)-Tetra-*O*-Acetyl-d-Mannopyranoside

**Figure 6:**
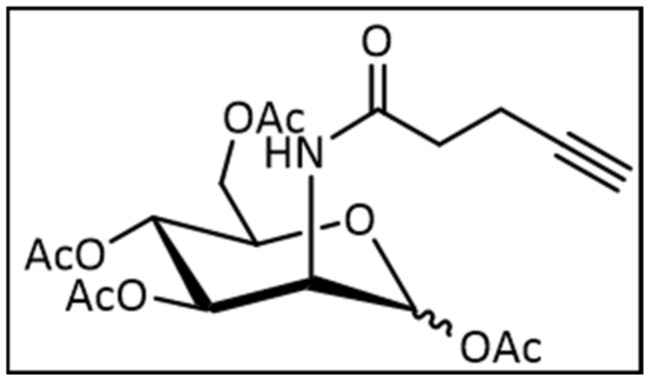
Structural formula of 2-(*N*-4-Pentynoyl)-2-Deoxy-(1,3,4,6)-Tetra-*O*-Acetyl-d-Mannopyranoside.

Previous alkyne mannose (230 mg, 887 µmol, 1.00 eq.) was suspended in 3 mL pyridine and cooled at 0 °C. Acetic anhydride (839 µL, 8.87 mmol, 10.0 eq.) was added and the mixture was stirred at ambient temperature for 18 h. The mixture was diluted with DCM (150 mL) and the organic layer was washed with saturated NaHCO_3_ (30 mL) solution and brine (30 mL). The organic layer was dried over MgSO_4_ and the crude product was purified by column chromatography (Cy:EE 1:1). Yield: 87% (330 mg, 772 µmol). Figure 6 is the structural formula of 2-(*N*-4-Pentynoyl)-2-Deoxy-(1,3,4,6)-Tetra-*O*-Acetyl-d-Mannopyranoside.

The ^1^H-NMR of a major anomer (400 MHz, CDCl_3_) (Supplementary Figure S3): 6.11 (d, ^3^*J* = 9.1 Hz, 1H, N*H*), 6.04 (d, ^3^*J* = 1.8 Hz, 1H, *H*-1), 5.32 (dd, ^3^*J* = 10.2 Hz, ^3^*J* = 4.4 Hz, 1H, *H*-3), 5.22 (dd, ^3^*J* = 10.0 Hz, 1H, *H*-4), 4.67 (ddd, ^3^*J* = 9.3 Hz, ^3^*J* = 4.4 Hz, ^3^*J* = 1.8 Hz, 1H, *H*-2), 4.29–4.24 (m, 1H, *H*-6), 4.11–4.01 (m, 1H, *H*-5, *H*-6), 2.56–2.45 (m, 1H, *H*-2′, *H*-3′), 2.17 (s, 3H, Ac), 2.09 (s, 3H, Ac), 2.05 (s, 3H, Ac), 2.00 (s, 3H, Ac) ppm; ^13^C-NMR of a major anomer (100 MHz, CDCl_3_) (Supplementary Figure S4): 171.36 (*C*-1′), 170.67 (*C*O(O)-6), 170.23 (*C*O(O)-3), 169.77 (*C*O(O)-4), 168.29 (*C*O(O)-1), 91.78 (*C*-1), 82.66 (*C*-4′), 70.65 (*C*-5′), 70.26 (*C*-5), 68.97 (*C*-3), 65.56 (*C*-4), 62.12 (*C*-6), 49.37 (*C*-2), 35.47 (*C*-2′), 21.00-20.78 (4 × *C*H_3_) 15.13 (*C*-3′) ppm.

The ^1^H-NMR of a minor anomer: (400 MHz, CDCl_3_): 6.07 (d, ^3^*J* = 9.1 Hz, 1H, N*H*), 5.86 (d, ^3^*J* = 1.8 Hz, 1H, *H*-1), 5.16 (dd, ^3^*J* = 9.8 Hz, 1H, *H*-4), 5.04 (dd, ^3^*J* = 10.0 Hz, ^3^*J* = 4.0 Hz, 1H, *H*-3), 4.80 (ddd, ^3^*J* = 9.2 Hz, ^3^*J* = 4.0 Hz, ^3^*J* = 1.7 Hz, 1H, *H*-2), 4.29–4.24 (m, 1H, *H*-6), 4.11–4.01 (m, 1H, *H*-6), 4.80 (ddd, ^3^*J* = 9.7 Hz, ^3^*J* = 5.1 Hz, ^3^*J* = 2.5 Hz, 1H, *H*-5), 2.56–2.45 (m, 1H, *H*-2′, *H*-3′), 2.10 (s, 3H, Ac), 2.10 (s, 3H, Ac), 2.05 (s, 3H, Ac), 2.00 (s, 3H, Ac) ppm; ^13^C-NMR of a minor anomer (100 MHz, CDCl_3_): 171.81 (*C*-1′), 170.69 (*C*O(O)-6), 170.30 (*C*O(O)-3), 169.77 (*C*O(O)-4), 168.50 (*C*O(O)-1), 90.69 (*C*-1), 82.73 (*C*-4′), 73.58 (*C*-5), 71.46 (*C*-3), 70.65 (*C*-5′), 65.38 (*C*-4), 62.00 (*C*-6), 49.60 (*C*-2), 35.64 (*C*-2′), 21.00-20.78 (4 × *C*H_3_), 15.21 (*C*-3′) ppm. HRMS (ESI): *m/z* calculated for C_19_H_25_NO_10_ [M-Na^+^] 450.1371, measured 450.1365, (|Δ*m/z*| = 1.3 ppm).

### 4.2. Cell Viability and Apoptosis Assays

To examine cell viability and apoptosis, hMSC-TERT were seeded in a 96-well plate (1 × 10^3^ cells per well) to adhere at 37 °C and 5% CO_2_ overnight. Cells were treated with peracetylated *N*-azidoacetylglucosamine (Ac_4_GlcNAz), *N*-azidoacetylgalactosamine (Ac_4_GalNAz), *N*-azidoacetylmannosamine (Ac_4_ManNAz) (Jena Bioscience GmbH, Jena, Germany), or *N*-alkyneacetylmannosamine (Ac_4_ManNAl) in 20 µM or 50 µM, or with dimethyl sulfoxide (DMSO) (0.2% *v*/*v*) (AppliChem GmbH, Darmstadt, Germany) as a negative control for 72 h. Viability and apoptosis rates were assessed using the CellTiter-Glo Luminescent Cell Viability Assay and the Caspase-Glo 3/7 Assay, respectively (both Promega GmbH, Mannheim, Germany), according to the manufacturer’s instructions. Luminescence was measured with an Orion II Luminometer (Berthold Detection Systems, Pforzheim, Germany). Each reaction was performed in technical triplicates and luminescence values were averaged.

### 4.3. Live/Dead Assay

To evaluate the ratio of viable to dead cells, hMSC-TERT were seeded in a 24-well plate (4 × 10^4^ cells per well) to adhere at 37 °C and 5% CO_2_ overnight. For preparation of the click mixture, Dulbecco’s phosphate-buffered saline (DPBS) (AppliChem GmbH, Darmstadt, Germany) was supplemented with 50 µM, 75 µM or 100 µM CuSO_4_ (Merck KGaA, Darmstadt, Germany), 250 µM, 375 µM or 500 µM of tris(3-hydroxypropyl-triazolylmethyl)amine (THPTA) (Carl Roth GmbH + Co. KG, Karlsruhe, Germany), respectively, as well as 2.5 mM sodium ascorbate (Sigma-Aldrich Chemie GmbH, Munich, Germany) and incubated at 37 °C for 10 min. Meanwhile, cells were washed with DPBS followed by a treatment of one well with 70% (*v*/*v*) isopropanol (AppliChem GmbH, Darmstadt, Germany) for 5 min as a dead control. Cells were incubated with the mixture at 37 °C and 5% CO_2_ for 5 min and washed three times with DPBS. After staining by using the LIVE/DEAD Cell Imaging Kit according to the manufacturer’s instructions (Thermo Fisher Scientific, Waltham, MA, USA), cells were imaged with a LEICA fluorescence microscope (Leica Camera AG, Wetzlar, Germany) using a 10× objective.

### 4.4. Adipogenic Differentiation

For adipogenic differentiation, hMSC-TERT were seeded at a density of 2 × 10^5^ cells per well in 6-well plates, cultivated until confluence and incubated in adipogenic differentiation medium consisting of DMEM High Glucose, 10% FCS, 1 U mL^−1^ penicillin, 100 µg mL^−1^ streptomycin, 1 µM dexamethasone, 0.5 mM 3-isobutyl-1-methylxanthine (IBMX), 1 µg mL^−1^ insulin, and 100 µM indomethacin. After two weeks, the cells were harvested for RNA isolation and intracellular lipid droplet staining. Cells cultured in expansion medium served as negative controls and the medium was changed twice per week.

### 4.5. Osteogenic Differentiation

hMSC-TERT were differentiated into the osteoblastic lineage by seeding 2 × 10^5^ cells per well in 6-well plates, cultivated until confluence, and incubated in osteogenic differentiation medium consisting of DMEM High Glucose, 10% FCS, 1 U mL^−1^ penicillin, 100 µg mL^−1^ streptomycin (all Life Technologies GmbH), 50 µg mL^−1^ L-ascorbic acid-2-phosphate, 0.1 µM dexamethasone, and 10 mM β-glycerophosphate (all Sigma Aldrich GmbH). After two weeks, the cells were harvested for RNA isolation and mineralized matrix staining. Cells cultured in expansion medium served as negative controls and the medium was changed twice per week.

### 4.6. Histochemical Staining

For the detection of calcium hydrogen phosphate and hydroxylapatite in the extracellular matrix, hMSC-TERT were fixed in methanol, stained with alkaline Alizarin Red S (1% *w*/*v*) (Chroma-Schmidt GmbH, Stuttgart, Germany) for 2 min, and air dried. For the detection of intracellular lipid vesicles, adipogenic monolayer cultures were stained with Oil Red O solution (Merck, Darmstadt, Germany). Images were taken with a digital camera EX Z150 (Casio Computer Co, Tokyo, Japan).

### 4.7. RNA Isolation, Reverse Transcription and qPCR Analysis

For gene expression analyses, hMSC-TERT were seeded in 6-well plates (2 × 10^5^ cells per well) to adhere at 37 °C and 5% CO_2_ overnight and treated with 20 or 50 µM of Ac_4_ManNAz or Ac_4_ManNAl, or with DMSO (0.2% *v*/*v*) as negative control for 72 h. Total RNA was isolated by using NucleoSpin RNA II kit (Macherey-Nagel, Düren, Germany) according to the manufacturer’s instructions. For mRNA reverse transcription, 1 µg of total RNA was used for first strand cDNA synthesis with MMLV reverse transcriptase (Promega GmbH, Mannheim, Germany) in 25 µL total volume. cDNA was diluted 1:10, and 2 µL were used for real-time qPCR with the GoTaq qPCR Master Mix (Promega GmbH, Mannheim, Germany) in 20 µL total volume. Primers were obtained from Eurofins MWG Operon (Ebersberg, Germany) or from Biomers GmbH (Ulm, Germany) (Supplementary Table S1 for primer sequences and annealing temperatures). qPCR conditions were as follows: 95 °C for 3 min; 40 cycles: 95 °C for 10 s; respective annealing temperature for 10 s; 72 °C for 10 s; followed by melting curve analysis for specificity of the qPCR products by using the qPCR thermal cycler Professional Thermocycler Biometra (Analytik Jena AG, Jena, Germany). Each reaction was performed in technical triplicates and Ct values were averaged. Relative gene expression was calculated with the efficiency-corrected Ct model [58] using RPLP0 as the reference gene.

### 4.8. Fluorescence Imaging after Click Reaction on the Cell Surface

For the evaluation of metabolic glycoengineering, hMSC-TERT were seeded in a 24-well plate (1 × 10^4^ cells per well) to adhere at 37 °C and 5% CO_2_ overnight and incubated afterwards in growth medium with Ac_4_GlcNAz, Ac_4_GalNAz, Ac_4_ManNAz, or Ac_4_ManNAl at 20 µM or 50 µM, or with DMSO (0.2% *v*/*v*) as a negative control for 72 h. For the CuAAC click reaction, DPBS was supplemented with 50 µM CuSO_4_ (Merck KGaA, Darmstadt, Germany) and 250 µM THPTA (Carl Roth GmbH + Co. KG, Karlsruhe, Germany), as well as 2.5 mM sodium ascorbate (Sigma-Aldrich Chemie GmbH, Munich, Germany), and incubated at 37 °C for 10 min. Then, 20 µM alkyne-sulfo-Cy3 (Sigma-Aldrich Chemie GmbH, Munich, Germany) or azido-sulfo-Cy5 [59] were added and cells were treated with the mixture at 37 °C and 5% CO_2_ for 5 min after removal of the medium and one washing step with DPBS. After the click reaction, cells were washed three times with DPBS and incubated with growth medium for 30 min, 2 d, 4 d, or 6 d. For fluorescence imaging, cells were washed once with DPBS, incubated with a mixture of 2% (*v*/*v*) formaldehyde (Merck KGaA, Darmstadt, Germany) and 2.5% (*v*/*v*) glutaraldehyde (Sigma-Aldrich Chemie GmbH, Munich, Germany) at 4 °C for 10 min, washed three times with DPBS, and followed by an incubation with 300 nM 4′,6-diamidino-2-phenylindole (DAPI) (Sigma-Aldrich Chemie GmbH, Munich, Germany) in DPBS at room temperature for 10 min for nuclei staining and three washing steps with DPBS. Images were taken with a Zeiss Axio Observer 7 fluorescence microscope (Carl Zeiss AG, Oberkochen, Germany) using a 10× objective, while exposure time and gain for Cy3 or Cy5 fluorescence were kept constant. For qualitative comparison, brightness and contrast of Cy3/5-related fluorescence signals were adjusted according to the sample with the highest signal intensity. For indirect quantitation, grey values of unaltered Cy3/5 related fluorescence signals from three different visual fields per micrograph were measured using Fiji 1.53c (National Institutes of Health, Bethesda, MD, USA) [60] and averaged.

### 4.9. Data Processing

Numerical data were evaluated with Office Excel 2016 (Microsoft Corporation, Redmond, WA, USA) and graphs were created in Prism 7 (GraphPad Software, San Diego, CA, USA), while image processing was performed with Fiji [60] and its plug-in ScientiFig [61]. For statistical analysis, datasets were compared to DMSO-treated controls for significance in Office Excel 2016 using two-tailed paired *t*-tests, with which *p*-values less than 0.05 were considered to be statistically significant.

## Data Availability

All data presented in this study are available from the corresponding author on reasonable request.

## Supplementary Materials

The following are available online at https://www.mdpi.com/1422-0067/22/6/2820/s1.
