# Supplementary material for: Metabolic Glycoengineering in hMSC-TERT as a Model for Skeletal Precursors by Using Modified Azide/Alkyne Monosaccharides
Source: Int J Mol Sci. 2021 Mar 10;22(6):2820. doi: 10.3390/ijms22062820 (PMC7999278; doi:10.3390/ijms22062820)
Supplement: Supplementary file 1 [file ijms-22-02820-s001.pdf]

International Journal of
Molecular Sciences

MDPI

Supplementary Materials

Metabolic Glycoengineering in hMSC-TERT as a Model for Skeletal Precursors by Using Modified Azide/Alkyne Mono-Saccharides

Stephan Altmann [1], Jürgen Mut [2], Natalia Wolf [2], Jutta Meißner-Weigl [1], Maximilian Rudert [1], Franz Jakob [1], Marcus Gutmann [3], Tessa Lühmann [3], Jürgen Seibel [2] and Regina Ebert [1,*]

1. Bernhard-Heine-Center for Locomotion Research, University of Würzburg, Friedrich-Bergius-Ring 15, 97076 Würzburg, Germany; stephan.altmann@klh.de (S.A.); j-meissner-weigl.klh@uni-wuerzburg.de (J.M.-W.); m-rudert.klh@uni-wuerzburg.de (M.R.); f-jakob.klh@uni-wuerzburg.de (F.J.)
2. Institute of Organic Chemistry, University of Würzburg, Am Hubland, 97074 Würzburg, Germany; juergen.mut@uni-wuerzburg.de (J.M.); natalia.wolf@uni-wuerzburg.de (N.W.); juergen.seibel@uni-wuerzburg.de (J.S.)
3. Institute of Pharmacy and Food Chemistry, University of Würzburg, Am Hubland, 97074 Würzburg, Germany; Marcus.Gutmann@uni-wuerzburg.de (M.G.); tessa.luehmann@uni-wuerzburg.de (T.L.)

* Correspondence: r-ebert.klh@uni-wuerzburg.de; Tel.: +49-931-8031597

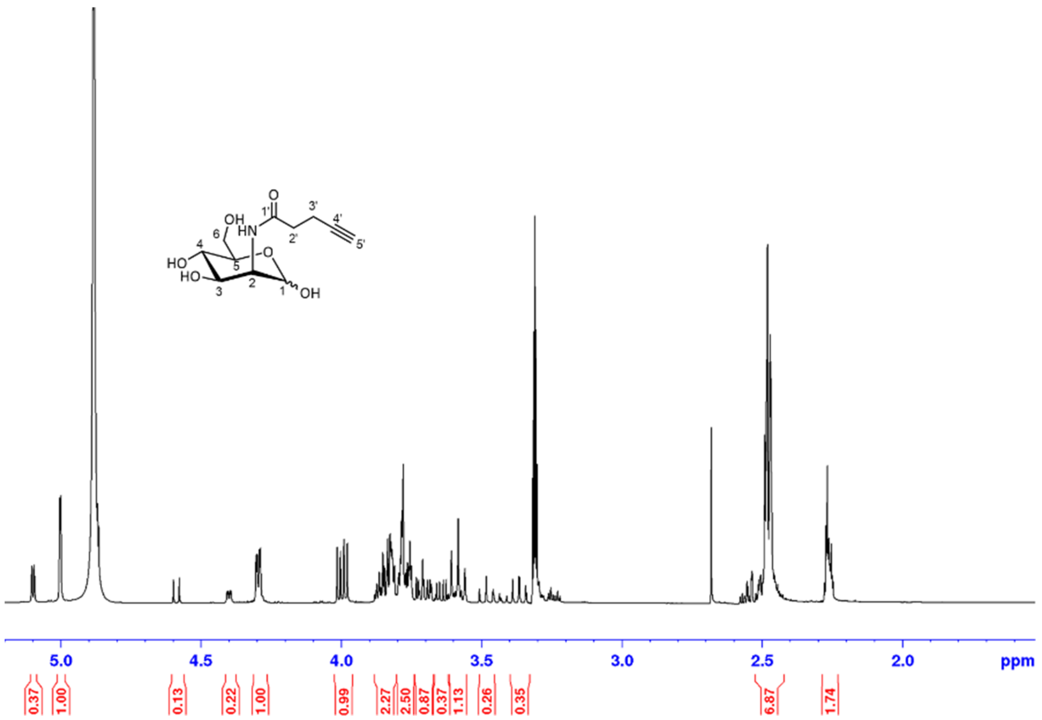

Supplementary Figure S1: ^{1}H-NMR spectrum of 2-(*N*-4-pentynoyl)-2-deoxy-D-mannopyranoside.

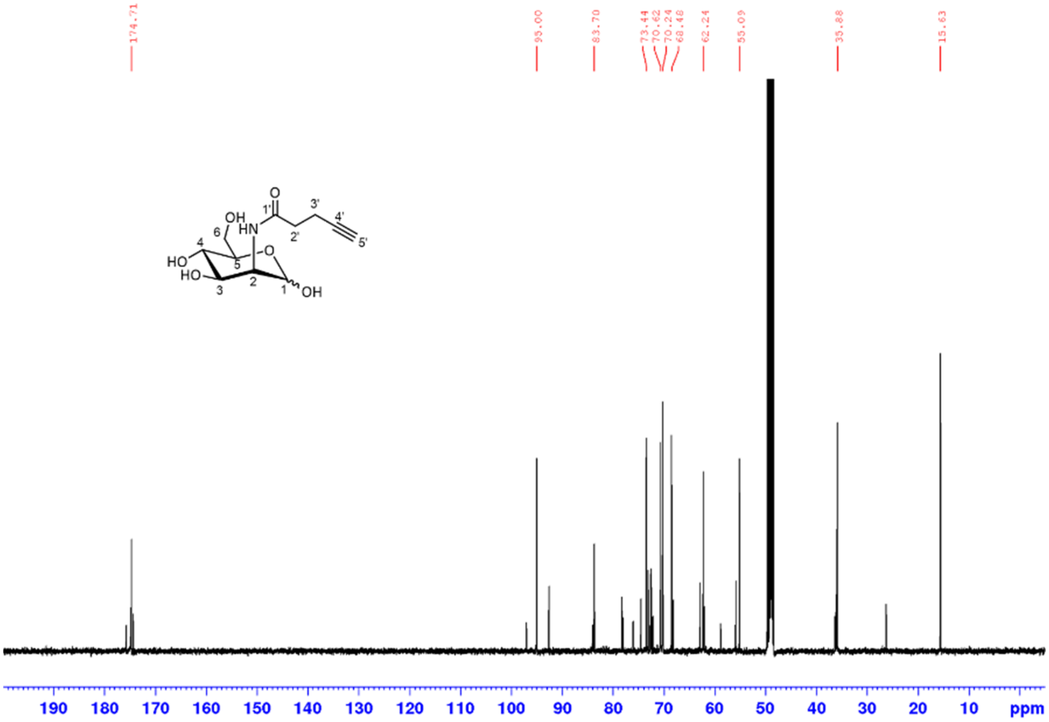

Supplementary Figure S2: ^{13}C-NMR spectrum of 2-(*N*-4-pentynoyl)-2-deoxy-D-mannopyranoside.

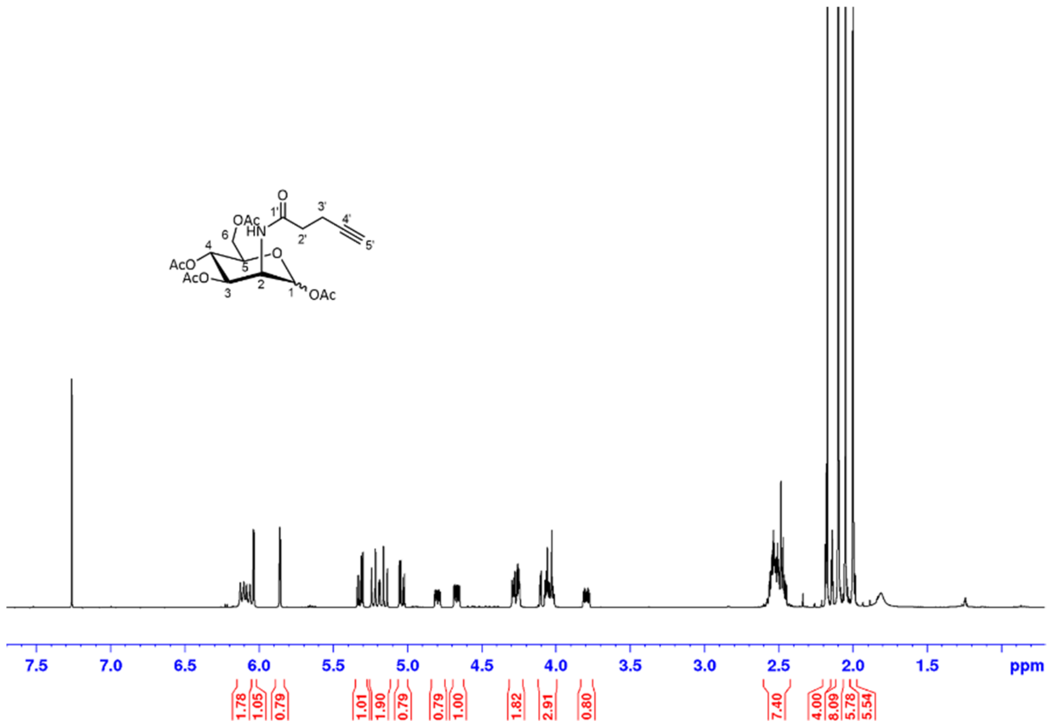

Supplementary Figure S3: ^{1}H-NMR spectrum of 2-(*N*-4-pentynoyl)-2-deoxy-(1,3,4,6)-tetra-*O*-acetyl-D-mannopyranoside.

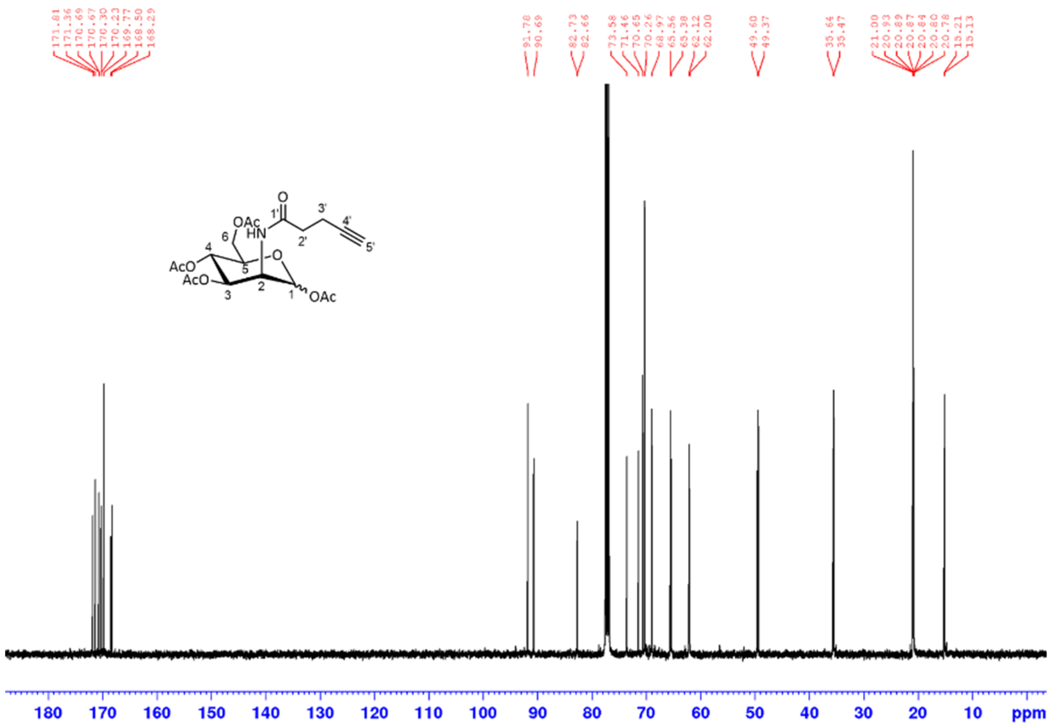

Supplementary Figure S4: ^{13}C-NMR spectrum of 2-(*N*-4-pentynoyl)-2-deoxy-(1,3,4,6)-tetra-*O*-acetyl-D-mannopyranoside.

Supplementary Table S1: Primers used for gene expression analyses. First sequence is sense, second is antisense.

Gene name	Gene ID	Sequence (5′ - 3′)	Annealing temp. [°C]	Product size [bp]
Acidic ribosomal protein P0	RPLP0	TGCATCAGTACCCCATTCTATCAT<br>AGGCAGATGGATCAGCCAAGA	60	122
Alkaline phosphatase	ALPL	GTACGAGCTGAACAGGAACAACG<br>CTTGGCTTTTCCTTCATGGTG	58	151
Bone gamma carboxyglutamate protein	BGLAP	TGACCACATCGGCTTTCAG<br>AAGGGGAAGAGGAAAGAAGG	60	126
Fatty acid binding protein	FABP	TACTGGGCCAGGAATTTGAC<br>GACACCCCCATCTAAGGTTATG	60	77
Peroxisome proliferator activated receptor gamma	PPARG	GTAAATCTGCGGGATGATGG<br>TGACCTGCGCAAAGTGTATC	60	200
Fibronectin	FN1	CTGAAAGACCAGCAGAGGCA<br>GTGTAGGGGTCAAAGCACGA	59	110
Integrin subunit alpha V	ITGAV	ATGTCACCTGGGGCATTCAG<br>TGTTCTTGAGGTGGCCG	59	154
Heparan sulfate proteoglycan	HSPG2	GGCAAGGACTTCATCAGCCT<br>ACTTGGATGGAACCTCTGCG	59	161
Laminin subunit gamma 2	LAMC2	CATTAGACGGCCTCCTGCAT<br>CGCAGTTGGCTGTTGATCTG	60	115
Thrombospondin 1	THBS1	TTTGGCCAGTCCAGCAG<br>AGAAAGGCCCGAGTATCCCT	60	108
Signal transducer and activator of transcription 1	STAT1	TGGGCTTCAGCAAGGAG<br>GTAGGGTTCAACCGCATGGA	60	167
Epidermal growth factor receptor	EGFR	AAGGCACGAGTAACAAGC<br>AGGGCAATGAGGACATAA	60	170
